# Combined Probiotics and Antioxidants Attenuate LPS-Induced Acute Intestinal Inflammation via Multi-Pathway Regulation in a Preventive Mouse Model

**DOI:** 10.3390/antiox15091201

**Published:** 2026-09-19

**Authors:** Jiaojiao Gao, Yong Tuo, Jishan An, Kailibinuer Abudukaiyoumu, Yuanyang Yi, Yuxia Yang, Tongjun Guo

**Affiliations:** 1 Institute of Feed Research, Xinjiang Academy of Animal Science, Urumqi 830011, China; gaojiaojiao@163.com (J.G.); t777yvx@126.com (Y.T.); 19220940025@163.com (J.A.); 17767554842@163.com (K.A.); 15022970169@163.com (Y.Y.); 2 Xinjiang Key Laboratory of Herbivorous Livestock Feed Biotechnology, Xinjiang Academy of Animal Science, Urumqi 830011, China

**Keywords:** microbial probiotics, antioxidants, intestinal barrier, gut microbiota, NF-κB pathway

## Abstract

**Objectives**: This study aimed to explore the combined regulatory effects of probiotics combined with bioactive compounds on intestinal immunity and mucosal barrier function. **Methods**: Fifty specific-pathogen-free (SPF) male Kunming mice (5–6 weeks old, weighing 20 ± 2 g) were randomly assigned to five groups (*n* = 10 per group): blank control (C), lipopolysaccharide-induced model (LPS), probiotic (PB), antioxidant (AO), and combined treatment (PB/AO). The C and LPS groups received daily oral gavage of 0.5 mL sterile 0.9% saline. The PB group received a daily gavage of 1 × 10^8^ CFU/mL *Pediococcus acidilactici* lindner and *Lactobacillus plantarum*. The AO group was administered a daily gavage of berberine (30 mg/kg BW), wogonin (30 mg/kg BW), and sodium butyrate (200 mg/kg BW). The PB/AO group received a daily gavage combining the probiotic mixture (1 × 10^8^ CFU/mL) with the plant extracts (30 mg/kg BW berberine, 30 mg/kg BW wogonin, and 200 mg/kg BW sodium butyrate). Following a 14-day preventive intervention, all groups except C were intraperitoneally injected with LPS to induce inflammation. Indices of visceral organs, serum antioxidant and immune parameters, ileal histomorphology, gut microbiota composition, and the expression of key barrier- and inflammation-related genes were comprehensively evaluated. **Results**: LPS exposure increased liver and spleen coefficients. PB/AO treatment significantly reduced the LPS-induced elevation of liver coefficient to a level comparable to that of the C group; however, no significant improvement in spleen coefficient was observed. In terms of antioxidant and immune indices, all intervention groups increased the levels of SOD, GSH-Px, and T-AOC to varying degrees, and decreased the levels of MDA and inflammatory factors (TNF-α, IL-1β, IL-6, and DAO), with the PB/AO group showing the most significant improvement (*p* < 0.01). Ileal microbiome analysis showed that PB/AO treatment enriched Firmicutes, Lactococcus and Lactobacillus but decreased Proteobacteria, with Clostridia as signature taxa. Mechanistically, PB/AO suppressed the transcription of NF-κB pathway-related genes (TLR4, MyD88 and NF-κB) and upregulated the expression of tight junction proteins (ZO-1, Claudin-1 and Occludin), thereby strengthening the intestinal barrier. **Conclusions**: In conclusion, co-administration of microbial probiotics and antioxidants achieves optimal protection against LPS-induced intestinal inflammation by jointly driving anti-inflammatory and antioxidative responses, modulating gut microbiota, and reinforcing barrier integrity.

## 1. Introduction

Inflammatory bowel disease (IBD) constitutes a substantial global health challenge, defined by persistent, chronic inflammation of the intestinal tract. Affecting more than 6.8 million individuals worldwide, the disease burden is particularly pronounced in industrialized nations. Epidemiological investigations have established that ultra-processed foods—ranging from extruded snacks and fried items to refined grains and sugar-sweetened beverages—are independent risk factors for the onset of IBD [1]. Beyond the hallmark symptoms of chronic abdominal pain and debilitating diarrhea, patients with severe IBD face life-threatening complications, including intestinal perforation and colorectal cancer [2]. While current therapeutic strategies offer symptomatic relief, they are frequently limited by adverse effects and modest long-term efficacy. This critical gap underscores the urgent need to investigate natural bioactive compounds as safer alternatives to prevent and attenuate intestinal inflammation. In the present study, we employed a single intraperitoneal injection of lipopolysaccharide (LPS) to establish an acute intestinal inflammatory stress model, which recapitulates the early endotoxin-triggered inflammatory cascade and allows for rapid evaluation of the preventive effects of candidate interventions on acute mucosal injury.

The etiology of IBD is characterized by a multifactorial interaction involving genetic predisposition, environmental factors, and immune system dysregulation [3]. Key pathophysiological mechanisms include the compromise of intestinal barrier integrity and the anomalous activation of inflammatory cascades. Specifically, Lipopolysaccharide (LPS), an endotoxin derived from Gram-negative bacteria, engages with receptors to trigger TLR4-dependent signaling. This process drives MyD88-mediated NF-κB activation, resulting in the excessive production of proinflammatory cytokines, such as TNF-α and IL-1β, which collectively perpetuate a vicious cycle of mucosal damage [4]. Crucially, the gut microbiota serves as a fundamental regulator of host immunity and barrier function [5,6]. In IBD patients, there is a marked reduction in beneficial bacteria, particularly Akkermansia (Verrucomicrobia) and the Firmicutes phylum, which are essential for maintaining homeostasis. In contrast, the expansion of pro-inflammatory Proteobacteria disrupts microbial balance, thereby exacerbating the inflammatory trajectory of the disease [7,8]. The acute LPS challenge model employed in this study shares key pathophysiological features with the acute exacerbation phase of IBD, including excessive TLR4/NF-κB activation, oxidative burst, and disruption of epithelial barrier integrity. However, it is important to distinguish this model from chronic IBD. In humans, IBD is a relapsing-remitting chronic disease driven by complex interactions between genetic susceptibility, environmental factors, and dysregulated immune responses. While LPS is not the primary cause of IBD, it is well established that gut barrier dysfunction in IBD patients leads to increased translocation of luminal LPS into the intestinal mucosa, where it activates TLR4 signaling and perpetuates inflammation. Increased serum LPS levels have been documented in IBD patients during active flares, and LPS has been shown to induce inflammatory cytokine production in IBD intestinal explants. Thus, while the acute LPS challenge model does not recapitulate the full complexity of chronic IBD, it provides a reproducible and controlled platform for studying the acute inflammatory pathways that are also activated in IBD flare-ups and for screening candidate interventions with potential anti-inflammatory and barrier-protective properties.

*Pediococcus acidilactici* lindner and *Lactobacillus plantarum*, together with the bioactive compounds berberine and wogonin, and the metabolite sodium butyrate, have garnered attention in IBD management due to their potential anti-inflammatory, antioxidant, and gut microbiota-modulating capabilities [9]. These substances may exert effects through multiple pathways, including modulating immune responses, attenuating oxidative stress, restoring gut microbial balance, and enhancing intestinal barrier function [10,11]. Moreover, a wealth of literature indicates that probiotic blends function via multifaceted mechanisms. These encompass immunomodulation, the re-establishment of intestinal microbial equilibrium, and the modulation of metabolic signaling, specifically targeting NLRP3 inflammasome activity and the AMPK/mTOR pathway [12]. Wogonin, a natural flavonoid, has been shown to inhibit TLR4/MyD88/NF-κB signaling, reduce pro-inflammatory cytokine secretion, and upregulate tight junction protein expression. Berberine, the principal active alkaloid from Coptis, upregulates antioxidant enzymes (SOD, CAT), scavenges reactive oxygen species, and mitigates oxidative damage [13]. Sodium butyrate, a short-chain fatty acid metabolite, increases tight junction protein expression (occludin, ZO-1), decreases intestinal permeability, and limits bacterial endotoxin translocation into the bloodstream [14]. However, systematic comparative studies on the mechanistic differences between probiotics and these bioactive compounds remain scarce, and evidence supporting combined probiotic–bioactive compound strategies based on complementary mechanisms is still limited. In this study, we systematically compared the efficacy of a composite probiotic, a bioactive compound mixture, and their combination in alleviating LPS-induced enteritis. We comprehensively evaluated TLR4/MyD88/NF-κB pathway activity, oxidative stress, gut microbiota composition, and intestinal barrier integrity to elucidate the multidimensional mechanistic differences among the three intervention strategies. We hypothesized that the combination of probiotics (*Pediococcus acidilactici* lindner and *Lactobacillus plantarum*) and antioxidants (wogonin, berberine, and sodium butyrate) would produce superior protective effects compared with either agent alone through complementary mechanisms: (1) the probiotic components would directly modulate gut microbiota composition and enhance intestinal barrier function via colonization and competitive exclusion; (2) the antioxidant compounds would suppress TLR4/MyD88/NF-κB signaling and reduce oxidative stress; and (3) the combination would achieve enhanced efficacy through coordinated, multi-pathway regulation of inflammatory signaling, oxidative defense, and tight junction integrity. This study aims to clarify potential combined or additive effects on key biological pathways, reveal the interplay among gut microbiota, metabolites, and host barrier function, and identify the optimal intervention model (monotherapy or combination) and its targets for preventing acute inflammatory injury in the intestine.

## 2. Materials and Methods

### 2.1. Experimental Materials

*Pediococcus acidilactici* Lindner and *Lactobacillus plantarum* were purchased from Henan Provincial Industrial Microbial Strains Engineering Technology Research Center. Berberine hydrochloride and wogonin (purity ≥ 98%) were obtained from Chengdu Mansite Bio-Technology Co., Ltd. (Chengdu, China). Sodium butyrate was purchased from Shanghai Yuanye Bio-Technology Co., Ltd. (Shanghai, China). Lipopolysaccharide (LPS) was obtained from Beijing Solarbio Science & Technology Co., Ltd. (Beijing, China). Specific pathogen-free (SPF) male Kunming mice were supplied by the Animal Experiment Center of Xinjiang Medical University.

### 2.2. Animal Handling, Diets, and Experimental Design

Fifty male SPF Kunming mice (5–6 weeks, 20 ± 2 g) were housed under controlled conditions (25 ± 2 °C, 60% humidity, 12 h light/dark) with free access to food and water. After a 7-day acclimation period, mice were randomly divided into 5 groups (*n* = 10 per group). The groups were designated as follows: blank control (C), lipopolysaccharide-induced model (LPS), probiotic (PB), antioxidant (AO), and combined treatment (PB/AO). The C and LPS groups received daily oral gavage of 0.5 mL sterile 0.9% saline. The PB group received a daily gavage of 1 × 10^8^ CFU/mL *Pediococcus acidilactici* lindner and *Lactobacillus plantarum* (1:1, volume ratio). The AO group was administered a daily gavage of berberine (30 mg/kg BW), wogonin (30 mg/kg BW), and sodium butyrate (200 mg/kg BW). The PB/AO group received a daily gavage combining the probiotic mixture (1 × 10^8^ CFU/mL) with the plant extracts (30 mg/kg BW berberine, 30 mg/kg BW wogonin, and 200 mg/kg BW sodium butyrate). All groups were gavaged once daily for 14 days. On day 14, mice in LPS, PB, AO, and PB/AO groups received an intraperitoneal injection of LPS (10 mg/kg BW) to establish an acute inflammatory stress model, while the C group received sterile saline. 4 h post-injection, mice were euthanized, and blood, ileal tissues, and ileal contents were collected for analyses. The protocol was approved by the Animal Ethics Committee of the Feed Research Institute, Xinjiang Academy of Animal Husbandry Sciences (Approval No. a2024-1206).

### 2.3. Measurement of Indicators

#### 2.3.1. Growth and Organ Indices

The initial body weight of each mouse was recorded prior to the formal experiment. On day 14 of the experimental period, the final body weight was recorded, followed by intraperitoneal injection of LPS or sterile saline according to the group allocation. Four hours post-injection, mice were euthanized and dissected. The heart, liver, spleen, lungs, and kidneys were aseptically excised, rinsed with pre-chilled sterile saline to remove residual blood, blotted dry with filter paper, and immediately weighed using an analytical balance with 0.1 mg precision (BSA124S, Sartorius, Göttingen, Germany). The organ indices were calculated using the following formula:

Organ Index (mg/10 g) = {Organ Weight (mg)/Body Weight (g)} × 10

#### 2.3.2. Plasma Biochemistry, Antioxidant and Immune Indexes

Blood samples were collected from the retro-orbital sinus of mice into heparin sodium-coated tubes and centrifuged at 3000 r/min for 15 min at 4 °C. The plasma supernatant was aliquoted and stored at −80 °C until further analysis. For plasma biochemical parameters, total protein (TP), total cholesterol (TC), triglycerides (TG), blood urea nitrogen (BUN), glucose (GLU), alanine aminotransferase (ALT), aspartate aminotransferase (AST), and alkaline phosphatase (ALP) were measured using an automatic biochemical analyzer (AU480, Beckman Coulter, Inc., Brea, CA, USA) with the manufacturer’s proprietary reagents. All measurements were performed strictly in accordance with the instrument’s standard operating procedures. For inflammatory cytokines, plasma levels of interleukin-1β (IL-1β), interleukin-6 (IL-6), and tumor necrosis factor-α (TNF-α) were determined using enzyme-linked immunosorbent assay (ELISA) kits (Beijing Huaying Biotechnology Institute, Beijing, China). Absorbance was read at 450 nm using a DR-200BS microplate reader (Huawei Delang, Wuxi, China). For oxidative stress markers and intestinal barrier function, plasma levels of superoxide dismutase (SOD), glutathione peroxidase (GSH-Px), total antioxidant capacity (T-AOC), malondialdehyde (MDA), and diamine oxidase (DAO) were measured using colorimetric assay kits (Beijing Huaying Biotechnology Institute, Beijing, China). All assays were performed according to the manufacturer’s instructions. Each sample was analyzed in duplicate (technical replicates), and the mean value of the two measurements was calculated for each mouse. These individual mouse means (n = 10 per group, representing biological replicates) were then used for all subsequent statistical comparisons. The intra-assay coefficient of variation for technical duplicates was <5% for all assays, confirming measurement precision.

Normalization procedure: To eliminate inter-sample variations in plasma protein content, the activities of SOD, GSH-Px, and T-AOC, as well as the levels of MDA and DAO, were normalized to the total protein concentration of each plasma sample, which was determined using a BCA protein assay kit (Beijing Huaying Biotechnology Institute, Beijing, China). Cytokine concentrations (TNF-α, IL-1β, and IL-6) were also expressed as pg/mg protein. The final results for all antioxidant, oxidative stress, inflammatory, and barrier-related parameters are therefore reported as units per mg of protein (U/mg protein, nmol/mg protein, or pg/mg protein, as appropriate).

#### 2.3.3. Histological Analysis

Immediately after collection, ileal tissues were fixed in 4% paraformaldehyde in the dark for 24 h, dehydrated in graded ethanol, cleared in xylene, and embedded in paraffin. The paraffin-embedded tissues were sectioned at a thickness of 3 μm and stained with hematoxylin and eosin (H&E). The stained sections were observed and images were captured under an optical microscope (Olympus BX53, Tokyo, Japan). For each section, at least three complete and well-oriented villus–crypt units were randomly selected. ImageJ software (version 1.54f; National Institutes of Health, Bethesda, MD, USA) was employed to quantify villus height and crypt depth. Villus height was measured from the villus tip to the villus–crypt junction. Crypt depth was measured from the crypt opening (intestinal luminal surface) vertically along the crypt axis to the crypt base (the basement membrane at the bottom of the crypt). The mean values of villus height, crypt depth, and the ratio of villus height to crypt depth (V/C) were calculated per mouse, and group means were derived from individual mouse means. All measurements were performed blindly with respect to group allocation by a single trained observer to minimize bias.

#### 2.3.4. Determination of Gut Microbiota Composition

After the experiment, mice were dissected, and the abdominal cavity was opened to isolate the ileal segments. The ileal segments were cut longitudinally, and the luminal contents were gently scraped, immediately frozen in liquid nitrogen, and stored at −80 °C until further analysis. Ileal contents were used for subsequent 16S rRNA gene sequencing. Shanghai BioProfile Technology Co., Ltd. (Shanghai, China) performed 16S rRNA gene sequencing on ileal contents. Microbial DNA was extracted using a Tiangen Biotech kit (Tiangen Biotech (Beijing) Co., Ltd., Beijing, China). Libraries were constructed from purified amplicons for PE 2 × 300 bp sequencing on an Illumina MiSeq PE300 system (Illumina, Inc., San Diego, CA, USA). For taxonomy, ASV sequences were aligned to the Greengenes database via the QIIME 2 feature-classifier plugin. Differential abundance of bacterial taxa was identified using ANCOM. Alpha and beta diversity indices were calculated using the QIIME 2 core-diversity plugin to assess species richness, evenness, and community structure variance.

#### 2.3.5. Quantitative Real-Time PCR (qRT-PCR) Analysis

Total RNA was isolated utilizing a Trizol kit (TransGen, Beijing, China), with subsequent purity and concentration assessments performed via spectrophotometry (Thermo Fisher Scientific, Waltham, MA, USA). The RNA was then reverse-transcribed into cDNA using a transcription kit. Quantitative PCR amplification was carried out on a Roche LightCycler 96 system in a 20 µL reaction mixture containing 10 µL of PerfectStart Green qPCR SuperMix, 10 ng of cDNA template, and 0.2 µM each of forward and reverse primers. The thermocycling protocol consisted of an initial denaturation at 95 °C for 30 s, followed by 40 cycles of 95 °C for 10 s, 60 °C for 15 s, and 72 °C for 10 s. All samples were analyzed in triplicate, and relative gene expression levels were calculated using the 2^−∆∆Ct^ method. Primer sequences for the target genes and the internal reference (β-actin) were designed based on NCBI mRNA sequences and synthesized by Prime Genetics (Table 1).

### 2.4. Statistical Analysis of Data

All data were organized using Microsoft Excel 2016 and analyzed using SPSS 26.0 (IBM Corp., Armonk, NY, USA) and R version 4.2.0 (R Core Team, Vienna, Austria). Statistical analyses were performed using the mean values derived from each biological replicate (*n* = 10 mice per group). Technical replicates (duplicate measurements per sample) were averaged prior to statistical testing and served as quality control to minimize analytical variability. All group comparisons were therefore based on biological variation, with analytical variation minimized through averaging.

Prior to parametric analysis, the normality of data distribution within each group was assessed using the Shapiro–Wilk test, and homogeneity of variances was evaluated using Levene’s test. For data meeting both normality (Shapiro–Wilk, *p* > 0.05) and homogeneity of variance (Levene’s, *p* > 0.05) assumptions, one-way analysis of variance (ANOVA) was performed. When the overall ANOVA was significant, Tukey’s honestly significant difference (HSD) post hoc test was used for all pairwise multiple comparisons, with the family-wise error rate (FWER) controlled at α = 0.05. For data that violated normality or homogeneity assumptions even after appropriate transformation, the non-parametric Kruskal–Wallis test was employed, followed by Dunn’s post hoc test with Bonferroni correction.

Statistical significance was set at *p* < 0.05, and highly significant differences were defined as *p* < 0.01. All reported *p*-values for multiple comparisons are adjusted values unless otherwise specified.

## 3. Results

### 3.1. Growth and Organ Indices

Table 2 illustrates that the initial and final body weights of the mice remained statistically comparable across all treatment groups (*p* > 0.05). Relative to the C group, the LPS challenge induced a substantial elevation in the liver index (*p* < 0.01) and a notable increase in the spleen index (*p* < 0.05). For the liver index, the PB/AO group demonstrated a highly significant reduction compared to the LPS group (*p* < 0.01) and was restored to a level comparable to that of the C group (*p* > 0.05). In contrast, for the spleen index, although LPS challenge significantly increased this parameter (*p* < 0.05 vs. C), none of the intervention groups (PB, AO, or PB/AO) showed a statistically significant reduction compared to the LPS group (*p* > 0.05), indicating that the treatments did not effectively reverse LPS-induced spleen enlargement within the experimental timeframe. No further significant variances were detected in the remaining parameters (cardiac, pulmonary, and renal indices) among the groups (*p* > 0.05).

### 3.2. Section of the Small Intestinal Tissue of Mice

**Figure 1:**
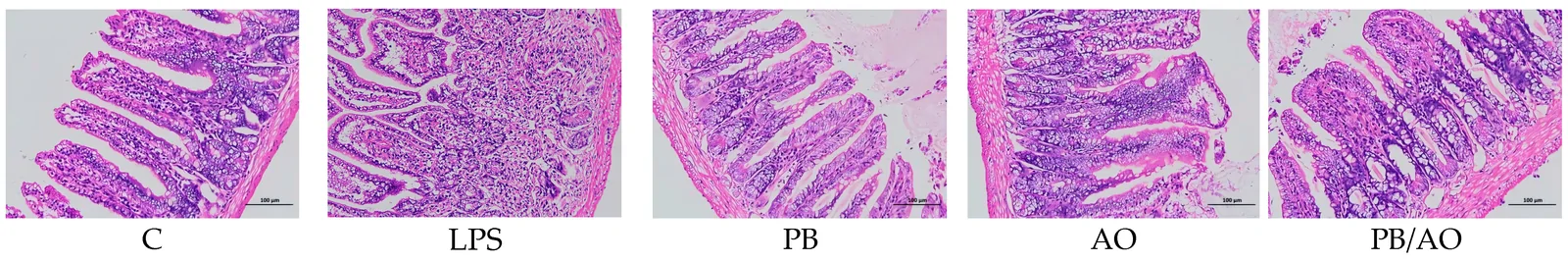
Histological images of ileal tissues collected from mice in different treatment groups.

As shown in Figure 1, the C, PB, and PB/AO groups displayed well-organized villi with intact epithelial integrity, whereas the LPS group exhibited marked villous blunting, epithelial desquamation, and architectural disruption. The AO group showed intermediate histological features, with partial preservation of villus structure. As summarized in Table 3, no statistically significant differences in villus length were detected among the five groups (*p* > 0.05). Compared with the C group, the LPS group had a significantly increased crypt depth and a significantly decreased villus-to-crypt (V/C) ratio (*p* < 0.01). The crypt depth in the PB, AO, and PB/AO groups was significantly reduced compared with the LPS group (*p* < 0.01), with the PB group exhibiting the lowest values among the intervention groups. The V/C ratio in the PB/AO group was significantly higher than that in the LPS group (*p* < 0.01) and was restored to a level comparable to the C group (*p* > 0.05). Although the PB/AO group showed a numerically higher V/C ratio than the PB and AO groups, no statistically significant differences were detected among the three intervention groups (*p* > 0.05).

### 3.3. Serum Biochemical Indices

Table 4 illustrates that relative to the C group, the LPS group manifested a highly significant elevation in BUN levels (*p* < 0.01) alongside a marked reduction in GLU levels (*p* < 0.01). In contrast to the LPS group, the AO group demonstrated a significant reversal of these trends, with BUN decreasing and GLU increasing significantly (*p* < 0.01); notably, these levels were restored to values comparable to the C group (*p* > 0.05). Although the PB and PB/AO groups also exhibited significant reductions in BUN compared to the LPS group (*p* < 0.05), their BUN levels remained significantly elevated above those of the C group (*p* < 0.05). Regarding GLU, neither the PB nor PB/AO groups differed significantly from the LPS group (*p* > 0.05), and both maintained levels significantly lower than the C group (*p* < 0.05). No statistically significant differences were detected in TP, TC, or TG across the five groups (*p* > 0.05), despite a numerical upward trend in TG observed in all LPS-treated groups compared to the C group. Of note, among the three intervention groups, the AO group exhibited superior efficacy in normalizing both BUN and GLU, achieving levels statistically indistinguishable from those of the C group (*p* > 0.05). In contrast, the PB and PB/AO groups, while significantly lowering BUN compared to the LPS group (*p* < 0.05), failed to restore BUN to the C level; similarly, neither PB nor PB/AO significantly improved GLU relative to the LPS group (*p* > 0.05). These results indicate that the antioxidant mixture (wogonin, berberine, and sodium butyrate) exerts a unique metabolic protective effect that is not enhanced, and perhaps partially attenuated, by the addition of probiotics.

### 3.4. Antioxidant and Immune Indexes

As indicated in Table 5, LPS induction resulted in a dramatic suppression of antioxidant markers (SOD, GSH-Px, T-AOC; *p* < 0.01) and a surge in inflammatory and damage markers (MDA, IL-1β, IL-6, TNF-α, DAO; *p* < 0.01) compared to the C group. Treatment with PB significantly restored SOD (*p* < 0.01) and GSH-Px (*p* < 0.05) levels while suppressing MDA (*p* < 0.01) and TNF-α (*p* < 0.05). The AO group displayed significant amelioration, evidenced by increased SOD, GSH-Px, and T-AOC (*p* < 0.01) and decreased MDA, TNF-α, and IL-6 (*p* < 0.01), with IL-1β and DAO also reduced significantly (*p* < 0.05). The PB/AO intervention proved highly effective, yielding extremely significant improvements across all measured indicators compared to the LPS group (*p* < 0.01).

### 3.5. Intestinal Microbiota Analysis via High-Throughput Sequencing

**Figure 2:**
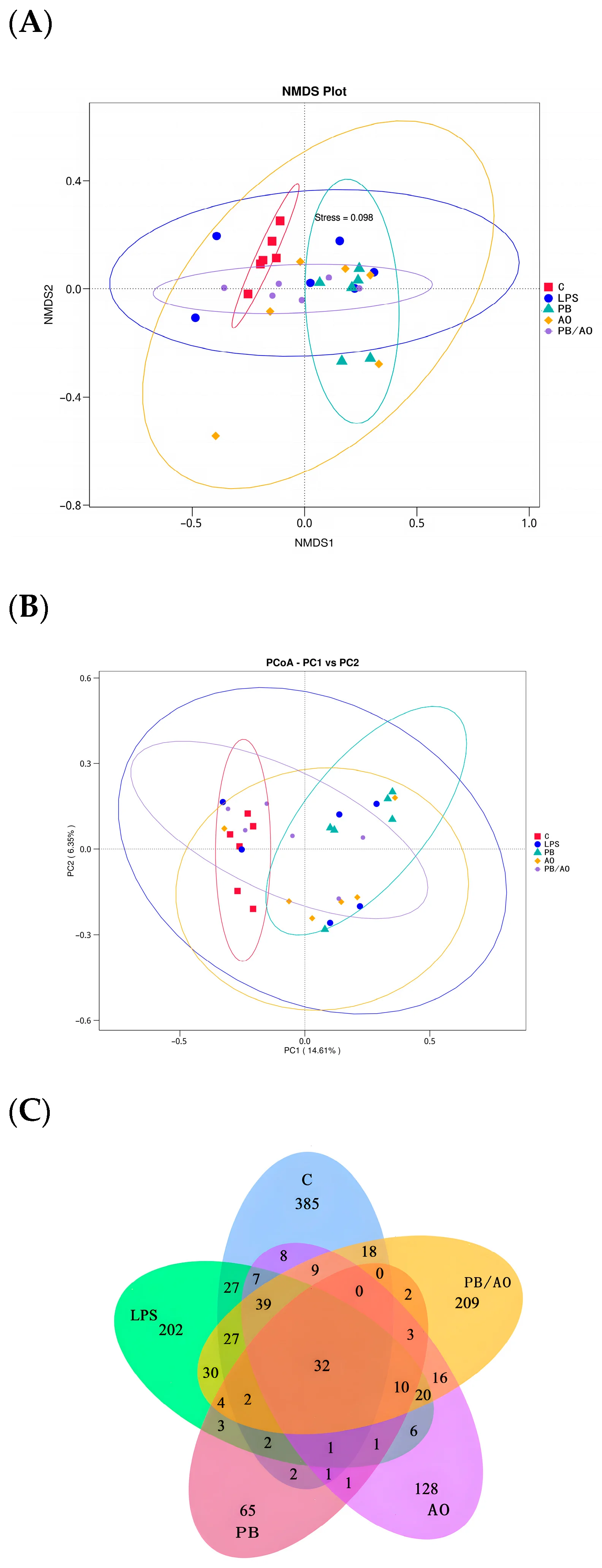
The mice exhibited a rich ileal microbiota diversity. (**A**) NMDS plots illustrating the differences in microbial community structure. (**B**) PCoA plots illustrating the differences in microbial community structure. (**C**) Venn diagram showing the overlap of the ASVs in the five groups. Statistical differences in β-diversity among groups were assessed by PERMANOVA (pseudo-F = 4.37, R^2^ = 0.28, *p* < 0.001) and ANOSIM (R = 0.43, *p* < 0.001) based on Bray–Curtis dissimilarities with 9999 permutations.

As shown in Figure 2A, the stress value of NMDS is 0.1, which is below 0.2, indicating that the NMDS data analysis is reasonable and that the C group and PB group are clearly separated. As shown in Figure 2B, in the PcoA analysis, PCo1 accounts for 14.61% and PCo2 accounts for 6.355%, and the C group and PB group are clearly separated.

Figure 2C illustrates the Venn diagrams generated from ASV analysis across different groups. A collective total of 1021 ASVs were identified, with 32 ASVs shared among all five groups. Regarding group-specific ASVs, the C group possessed 385 unique ASVs, followed by the PB/AO group with 209 and the LPS group with 202. The AO and PB groups contained 128 and 65 unique ASVs, respectively.

Table 6 illustrates that, relative to the C group, the PB and AO groups displayed numerically lower Chao1 and Observed_features indices. However, only the Observed_features index reached statistical significance (*p* < 0.05), whereas the Chao1 index did not show a significant difference (*p* = 0.061). No statistically significant disparities were detected in the remaining indices (Pielou_e, Dominance, Simpson, and Shannon) among the different groups (*p* > 0.05).

**Figure 3:**
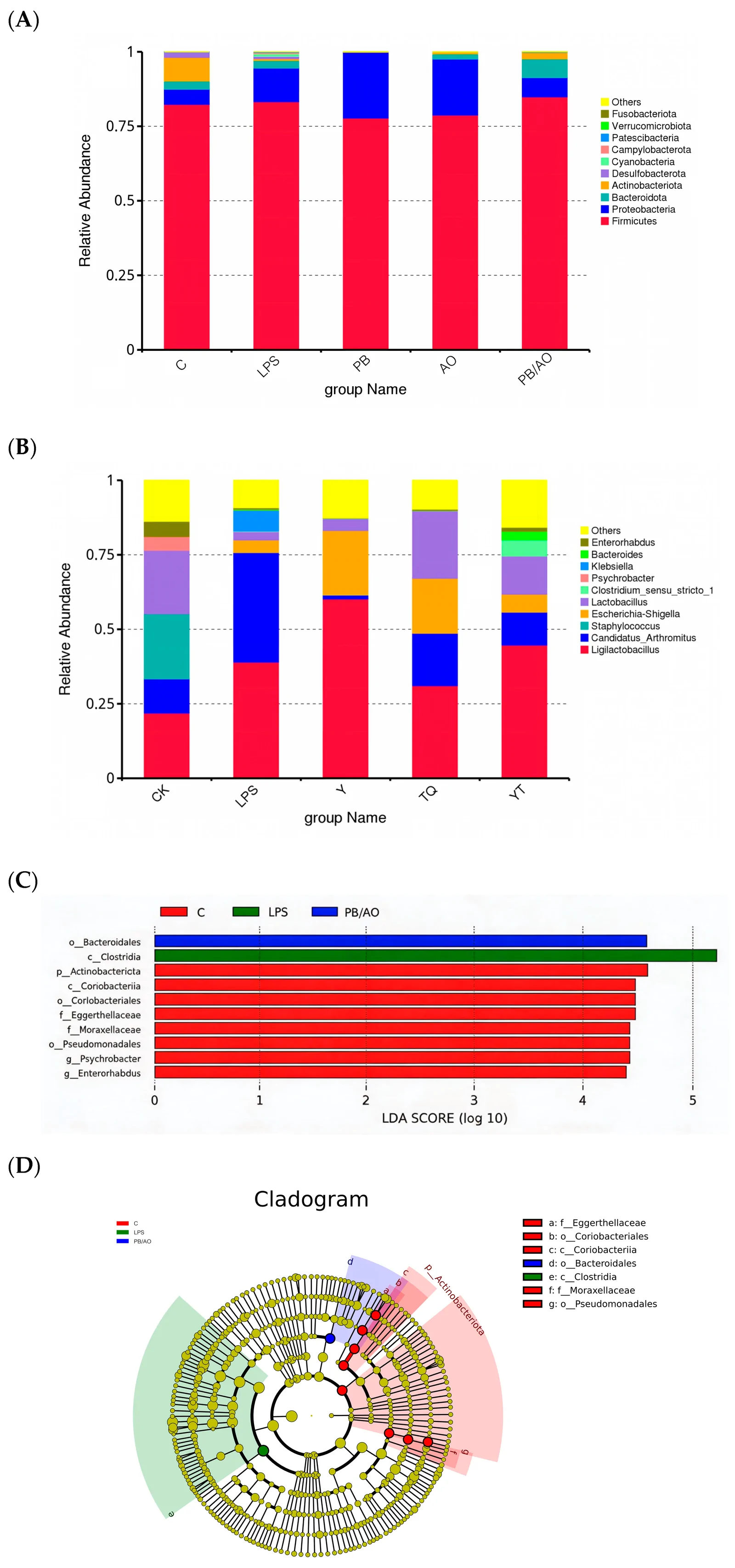
Composition of the ileal microbiota at the phylum (**A**) and genus (**B**) levels. (**C**) Histogram of the LDA score distribution and (**D**) cladogram (evolutionary branching diagram) derived from LEfSe analysis with a linear discriminant analysis (LDA) threshold of 4 (log_10_) and statistical significance set at *p* < 0.05 (Kruskal–Wallis test followed by Wilcoxon rank-sum test for pairwise comparisons).

Figure 3A illustrates the composition of the ileal microbiota at the phylum level, where Firmicutes and Proteobacteria were identified as the predominant phyla. In the compositional plots, Proteobacteria accounted for a larger proportion in the LPS group compared to the C group. In contrast, the PB/AO group displayed a reversed pattern, with Firmicutes occupying a greater proportion, whereas the AO and PB groups showed a higher relative abundance of Proteobacteria in their compositional profiles. Regarding the genus level, Figure 3B depicts the top 10 most abundant genera identified in the ileal microbiota of the experimental mice. The C group exhibited relatively higher genus abundances, with dominant genera including Ligilactobacillus, Lactobacillus, and Staphylococcus. The LPS group showed a higher proportion of Ligilactobacillus and Candidatus_Arthromitus, while the PB group displayed a relatively higher compositional abundance of Ligilactobacillus and Escherichia-Shigella. The AO and PB/AO groups showed dominant genera of Ligilactobacillus and Lactobacillus. Importantly, these descriptions are based on relative abundance proportions in the sequencing profiles; formal pairwise differential-abundance tests (e.g., ANCOM) were not performed for these specific taxa, and the observed differences should be interpreted as descriptive trends rather than statistically significant alterations.

LEfSe analysis integrates nonparametric Kruskal–Wallis and Wilcoxon rank-sum tests with linear discriminant analysis (LDA) effect size measurements to facilitate the identification of biomarkers. The outcomes of this analysis are illustrated in Figure 3C,D. At LDA = 4, the C group exhibits eight potential gut community markers: p_Actinobacteriota, c_Coriobacteria, o_Coriobacteriales, f_Eggerthellaceae, f_Moraxellaceae, o_Pseudomonadales, g_Psychrobacter, and g_Enterorhabdus. LPS has one potential gut community marker: o_Bacteroidales; PB/AO had one potential marker: c_Clostridia (class level).

### 3.6. Effects of Different Compound Preparations on Metabolites in Mouse Ileal Chyme

As shown in Table 7, no significant differences were observed among the groups treated with different compound formulations in terms of the metabolic products of chyme in the ileum of LPS-treated mice (*p* > 0.05).

### 3.7. NF-κB Signaling Pathway-Related Gene Expression

**Figure 4:**
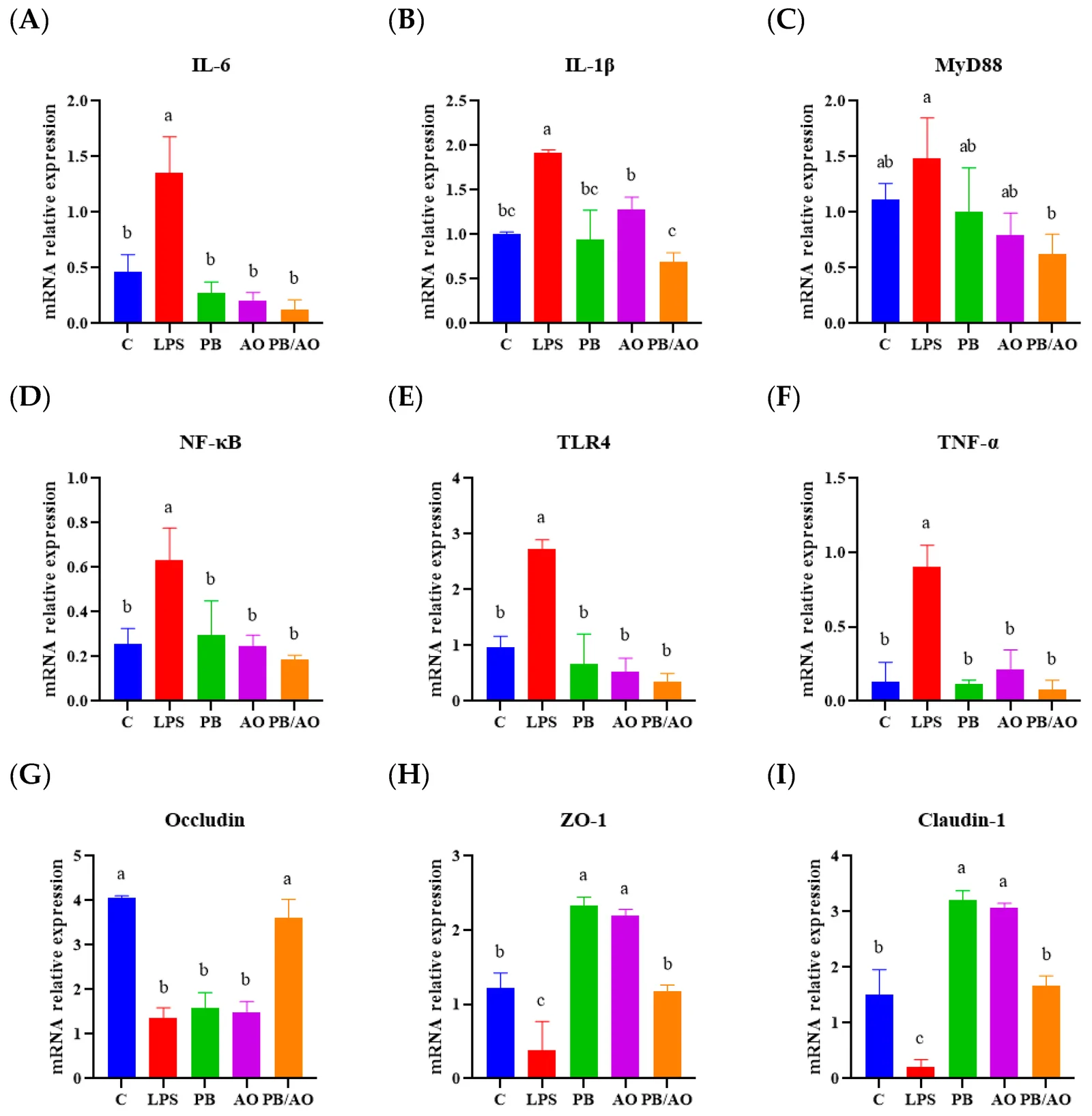
NF-κB signaling pathway components. (**A**) IL-6 mRNA, (**B**) IL-1βmRNA, (**C**) MyD88 mRNA, (**D**) NF-κB mRNA, (**E**) TLR4 mRNA, (**F**) TNF-α mRNA, (**G**) Occludin mRNA, (**H**) ZO-1 Mrna and (**I**) Claudin-1 mRNA. Within each row, values with different superscript letters indicate statistically significant differences. Lowercase letters (a, b, c) denote significant differences at *p* < 0.05. Groups sharing at least one identical letter (e.g., both contain “a”) are not significantly different from each other; groups sharing no common letter are significantly different at the corresponding threshold.

As shown in Figure 4, compared with the C group, the relative mRNA expression levels of NF-κB, TNF-α, IL-6, IL-1β, and TLR4 in the LPS group were all significantly increased (*p* < 0.05), while those of ZO-1, Claudin-1, and Occludin were all significantly decreased (*p* < 0.05); the relative mRNA expression level of MyD88 showed no significant difference. Compared with the LPS group, the relative mRNA expression levels of IL-6 and IL-1β in the PB, AO, and PB/AO groups were all significantly decreased (*p* < 0.05), and showed no significant difference compared with the C group (*p* > 0.05); the relative mRNA expression level of MyD88 in the PB/AO group was significantly decreased (*p* < 0.05), and showed no significant difference compared with the C group (*p* > 0.05); the relative mRNA expression levels of NF-κB, TLR4, and TNF-α in the PB, AO, and PB/AO groups were all significantly decreased (*p* < 0.05), and showed no significant difference compared with the C group (*p* > 0.05). Regarding tight junction proteins, the PB/AO group exhibited a significant upregulation of Occludin mRNA expression relative to the LPS group (*p* < 0.05), showing levels comparable to the C group (*p* > 0.05). For ZO-1 and Claudin-1, mRNA levels in the PB, AO, and PB/AO groups were all significantly elevated compared to the LPS group (*p* < 0.05). Notably, the PB and AO groups displayed significantly higher expression levels than the C group (*p* < 0.05), whereas the PB/AO group showed no significant difference from the C group (*p* > 0.05). No other significant differences were detected among the remaining groups (*p* > 0.05).

## 4. Discussion

Acute inflammatory stress induced by LPS shares key pathophysiological features with the acute exacerbation phase of IBD, including excessive TLR4/NF-κB activation, oxidative burst, and disruption of epithelial barrier integrity. Lipopolysaccharide (LPS), a potent endotoxin, not only induces structural damage to immune organs but also triggers excessive inflammatory responses through the overactivation of host immunity [15,16]. While probiotics and plant-derived bioactives have emerged as promising multi-target interventions for IBD, their combined effects and underlying mechanisms remain incompletely understood. This study proposed the core hypothesis of a multi-target combined protective mechanism mediated by the combined intervention of probiotics and antioxidants. It aimed to investigate whether the compound intervention could alleviate LPS-induced intestinal injury by inhibiting the TLR4/MyD88/NF-κB inflammatory pathway, upregulating tight junction proteins, and stabilizing intestinal flora homeostasis. The experimental indicator system of this study centered on mechanism verification and inter-group efficacy comparison, focusing on evaluating the regulatory effects of different intervention methods on inflammatory pathways and intestinal barrier function. Meanwhile, biochemical and microbiomic indicators were supplemented for exploratory analysis to comprehensively investigate the regulatory effect of the compound preparation on the overall physiological state of the organism. Accordingly, a compound preparation composed of two probiotics (*Pediococcus acidilactici* Lindner and *Lactobacillus plantarum*) and three plant active ingredients (wogonin, berberine, and sodium butyrate) was constructed, and its intervention efficacy against LPS-induced colitis was verified. The results showed that the compound preparation exhibited significantly superior protective effects compared with single-component interventions. Mechanistic studies confirmed that the compound preparation alleviated intestinal injury via multi-pathway combined regulation, including inhibiting the TLR4/MyD88/NF-κB inflammatory signaling pathway, upregulating tight junction protein expression to restore intestinal barrier integrity, and remodeling the homeostasis of ileal microbial community, with all the above regulatory effects significantly better than those of single treatment groups.

Previous investigations have established the individual benefits of probiotics and phytochemicals in both chronic IBD models and acute inflammatory models. For instance, mixed Lactobacillus supplementation effectively alleviates DSS-induced intestinal inflammation [17], while berberine and wogonin derivatives attenuate LPS-induced multi-organ injury through enhancement of antioxidant capacity [18]. Consistent with these observations, our study found that both single and combined interventions ameliorated LPS-induced organ damage and oxidative stress. Among the three intervention regimens, the combined intervention group (PB/AO group) demonstrated generally favorable effects across most endpoints related to inflammation and oxidative stress. This was evidenced at the organ and serological levels: the standardized liver index in the PB/AO group recovered to a level comparable to the C group, and antioxidant enzyme activities (SOD, GSH-Px, and T-AOC) were significantly elevated. However, it should be noted that the protective effects were not uniform across all parameters. For instance, the AO group (antioxidants alone) restored serum BUN and GLU to levels comparable to the C group, whereas the PB/AO group did not achieve the same degree of normalization (*p* < 0.05 vs. C for PB/AO), suggesting that the antioxidant mixture exerts a distinct metabolic protective effect that is not enhanced by the addition of probiotics. Furthermore, for intestinal histological parameters such as the villus-to-crypt ratio, the differences between PB/AO and the single-agent groups (PB or AO) did not reach statistical significance (*p* > 0.05 for multiple comparisons). Therefore, while the PB/AO combination showed numerical advantages in most gut-specific endpoints, its superiority was not universal, and we have revised our interpretation to reflect this nuanced pattern. These results indicate that the combined effects of viable probiotics and antioxidant compounds are predominantly manifested at the intestinal mucosal interface, where microbial and chemical signals coordinately participate in and reinforce the regulation of intestinal barrier function and local immune homeostasis.

Furthermore, the PB/AO group exhibited the most significant reductions in MDA, DAO, and pro-inflammatory cytokines (IL-1β, IL-6, and TNF-α), further corroborating its central role in ameliorating intestinal barrier dysfunction and suppressing inflammatory cascades. However, no significant improvement in the spleen index was observed across any of the intervention groups. This differential response between the liver and spleen indices likely reflects the distinct dynamic characteristics of these two organs in response to LPS stimulation: the liver, serving as the primary site for endotoxin clearance and acute-phase protein synthesis, may respond more rapidly to nutritional interventions [19]; in contrast, the activation and proliferation of immune cells in the spleen may require a longer duration of intervention or recovery to manifest measurable changes in organ weight [20]. This 4 h post-intervention time point (optimized to capture the peak acute inflammatory cytokine response) may not be the optimal window for detecting structural or cellular alterations within the spleen.

The crosstalk between inflammatory signaling and intestinal barrier integrity is central to acute inflammatory injury and is also a hallmark of IBD pathogenesis [21]. The engagement of TLR4 by LPS initiates the MyD88/NF-κB signaling axis, which promotes the transcription of pro-inflammatory cytokines and simultaneously impairs the integrity of the physical barrier through the suppression of tight junction proteins, including claudins, occludin, and ZO-1. Each component of our combined preparation has been independently reported to modulate this network: *Pediococcus acidilactici* and *Lactobacillus plantarum* can stimulate gut-associated lymphoid tissue and promote regulatory T cell differentiation; wogonin and berberine inhibit NF-κB activation; and sodium butyrate exerts immunomodulatory effects via histone deacetylase inhibition [22]. However, the collective impact of these agents on the TLR4/MyD88/NF-κB pathway and its downstream effects on barrier function has not been systematically evaluated. In our study, LPS challenge significantly upregulated the mRNA expression of TLR4, MyD88, NF-κB, and the downstream cytokines TNF-α, IL-6, and IL-1β, while concurrently downregulating ZO-1, claudin-1, and occludin—confirming the disruption of both inflammatory and barrier homeostases. Remarkably, the PB/AO intervention reversed all these changes: TLR4/MyD88/NF-κB signaling was robustly suppressed, and the expression of tight junction proteins was restored to near-control levels. These results are consistent with the findings of Guo et al. [23], who demonstrated that Bacteroides-based microecologics alleviated LPS-induced downregulation of tight junction proteins, and Zhou et al. [24], who reported that wogonin treatment increased MUC2 and occludin protein levels in DSS-induced colitis. Furthermore, Dou et al. [25] showed that oral sodium butyrate in DSS-induced colitis mice effectively restored colon length, reduced epithelial degeneration and ulceration, and markedly decreased inflammatory cell infiltration in the spleen. Collectively, our data indicate that the PB/AO formulation exerts its anti-inflammatory effects by intercepting the TLR4/MyD88/NF-κB axis at multiple points, while simultaneously reinforcing the epithelial barrier. This combined action contributed to the generally favorable outcomes observed in the PB/AO group across most inflammatory and barrier-related endpoints. However, given that formal interaction analysis did not demonstrate statistical synergy, the benefits of the combination should be interpreted as additive rather than synergistic.

Gut microbiota, as a key regulator of host immune responses and metabolic processes, plays an important role in shaping inflammatory states and maintaining intestinal barrier integrity. It is well documented that LPS stimulation disrupts gut microbial homeostasis, typically manifested as an expansion of pro-inflammatory phyla (e.g., *Proteobacteria*) and a depletion of beneficial commensal microbiota [26]. Consistent with these findings, our 16S rDNA sequencing analysis of ileal contents revealed that LPS treatment significantly increased the relative abundance of *Proteobacteria*. Notably, this LPS-induced dysbiosis was effectively reversed in the PB/AO combined intervention group, characterized by a specific enrichment of *Firmicutes* and a reduction in *Proteobacteria*; whereas in the single-component intervention groups (groups PB and AO), an increased abundance of opportunistic pathogens (*Escherichia-Shigella*) was observed. This unique microbial community restructuring, observed exclusively in the PB/AO group, suggests that the combined application of probiotics and antioxidants exerts a combined stabilizing effect on the ileal microbial ecosystem, an effect unattainable by any single component alone.

Specifically, the marked decrease in the compositional abundance of Escherichia-Shigella within the PB/AO group is of particular interest, as this genus is frequently associated with intestinal inflammation, mucosal damage, and IBD exacerbation. However, given the absence of formal differential-abundance statistics for this specific pairwise comparison, this observed trend should be interpreted as a preliminary observation rather than a confirmed inhibitory effect [27]. The suppression of its abundance reflects the potent inhibitory effect of the combined preparation against opportunistic pathogens. Concurrently, PB/AO intervention established *Lactobacillus* as the dominant genus. It is well documented that the enrichment of *Lactobacillus* facilitates the production of antimicrobial substances, promotes mucus secretion, and competitively excludes pathogens, thereby reinforcing intestinal barrier function and attenuating inflammatory responses [28,29]. Furthermore, LEfSe analysis identified the class c_Clostridia as a characteristic taxon enriched in the PB/AO group (Figure 3C,D). Notably, this taxon is classified at the class level, which encompasses a wide phylogenetic diversity. Although certain members belonging to this class have been previously reported to possess immunomodulatory properties, the taxonomic resolution of our current 16S rRNA sequencing (V3–V4 region) does not permit precise assignment to the genus Clostridium, nor does it allow functional inference regarding butyrate production. Therefore, while the enrichment of c_Clostridia in the PB/AO group supports the notion of a shifted microbial ecosystem in response to the combined intervention, the specific functional mediators or metabolic pathways involved (including those related to immune regulation) remain to be determined in future studies using metagenomic or metatranscriptomic approaches [30]. It is noteworthy that although we observed an enrichment of this taxon in the PB/AO group, the concentrations of SCFAs (acetate, propionate, and butyrate) in the ileal chyme did not differ significantly among groups. This discrepancy may be attributed to the early (4 h) time point of sampling, which may not allow sufficient accumulation of fermentation end-products in the ileal lumen, or to the rapid absorption and utilization of these metabolites by the host. Nevertheless, the enrichment of Clostridium in the PB/AO group further supports the notion that the combined intervention fosters a more resilient and immunologically balanced microbial community, even though direct changes in luminal SCFA levels were not detected in this acute model.

In summary, the present study demonstrates that a combinatory regimen of probiotics (*Pediococcus acidilactici* lindner and *Lactobacillus plantarum*) and plant-derived bioactive compounds (wogonin, berberine, and sodium butyrate) confers superior protection against LPS-induced acute colitis compared to its individual components. The protective mechanisms of this regimen are characterized by a multifaceted combined action: attenuation of local inflammation via inhibition of the TLR4/MyD88/NF-κB pathway, restoration of the intestinal epithelial barrier through the upregulation of tight junction proteins, and reversal of microbial dysbiosis by enriching beneficial taxa, including *Lactobacillus* and SCFA-producing *Clostridium*. These findings elucidate the combined mechanisms of probiotics and plant extracts at the intestinal mucosal interface, providing an experimental basis and therapeutic rationale for multi-target interventions in acute IBD exacerbations and other endotoxin-driven intestinal injuries.

A notable limitation of this study is the reliance on a single time-point measurement (4 h post-LPS). Although this time point was strategically chosen to capture the peak acute inflammatory cytokine response based on preliminary kinetic data and existing literature—and is optimal for assessing acute inflammatory and oxidative stress—it precludes the evaluation of the resolution phase and long-term protective effects. Furthermore, in the absence of targeted pathway-specific inhibition or genetic manipulation, the mechanistic interpretations presented here are hypothesis-generating rather than definitive.

Furthermore, while 16S rRNA gene sequencing (targeting the V3–V4 hypervariable region) effectively captured compositional shifts in the ileal microbial community, its taxonomic resolution is inherently constrained—particularly for lineages such as the class Clostridia, for which accurate assignment is often limited to higher ranks (phylum or class). Moreover, this approach does not reliably predict functional gene content, metabolic pathways, or specific effector molecules. This resolution gap is critically underscored by our SCFA data: although LEfSe analysis identified Clostridia as a signature taxon in the PB/AO group, no significant differences in acetate, propionate, or butyrate concentrations were detected among groups. This discrepancy likely reflects the inherent limitation of amplicon sequencing in inferring metabolic function. These constraints clearly delineate priorities for future research, which should incorporate longitudinal multi-time-point analyses (e.g., 2, 4, 12, 24, and 72 h post-LPS) and utilize pharmacological inhibitors (e.g., NF-κB inhibitors, TLR4 antagonists), gene-editing models, or rescue experiments to establish definitive causal links between the candidate pathways and the observed protective phenotypes.

## 5. Conclusions

In an LPS-induced acute intestinal inflammatory stress model of inflammatory bowel disease (IBD), we demonstrated that preventive administration of a combined preparation consisting of microbial probiotics (*Pediococcus acidilactici* lindner and *Lactobacillus plantarum*) and antioxidants (wogonin, berberine, and sodium butyrate) effectively prevented the development of intestinal inflammation. LPS challenge induced marked increases in liver and spleen indices, disruption of intestinal barrier integrity, oxidative stress, and robust release of pro-inflammatory cytokines—all of which were significantly reversed by the combined intervention (PB/AO). Notably, this combined regimen outperformed all single-agent treatments, highlighting its potential as a safe, effective, and economical nutritional strategy for IBD management.

## Figures and Tables

**Table 1:** qRT-PCR primer sequences.

Accession Number	Gene Names	Primer Sequence (5′-3′)
NM_007393.5	β-actin	F:AATCCTGCGGCATCCACGAAAC
		R:CAGCACCGTGTTGGCGTAGAG
NM_010851.3	MyD88	F:GCATGGTGGTGGTTGTTTCTG
		R:GAATCAGTCGCTTCTGTTGG
NM_008689.3	NF-κB	F:ACACTGGAAGCACGGATGAC
		R:TGTCTGTGAGTTGCCGGTCT
NM_016674.4	Claudin-1	F:TCCAGAAAGAAGCCAGATCC
		R:GTAGACATCGCCGTCAAAAG
NM_009386.2	ZO-1	F:GCCTAAAGCTGTTCCTGTGAGTCC
		R:ACCCCGCCGTTGCTGTTAAAC
NM_008756.2	Occludin	F:TGGCTGCCTTCTGCTTCATTGC
		R:GAACACCATCACACCCAGGATAGC
NM_021297.4	TLR4	F:GCACTGTTCTTCTCCTGCCT
		R:AGAGGTGGTGTAAGCCATGC
NM_008361.4	IL-1β	F:TGGTGTGTGACGTTCCCATT
		R:CAGCACGAGGCTTTTTTGTTG
NM_031168.2	IL-6	F:GGGACCCGAGTTACTACTTG
		R:CTGGGCTCTGCTATCCAAGG
NM_013693.3	TNF-α	F:CAAAATTCGAGTGACAAGCC
		R:TTGTCCCTTGAAGAGAACCT

**Table 2:** Effects of different composite formulations on the organ indices of LPS-challenged mice.

Items	Groups					SEM	*p*
	C (*n* = 10)	LPS (*n* = 10)	PB (*n* = 10)	AO (*n* = 10)	PB/AO (*n* = 10)		
Initial weight (g)	35.00	34.00	37.00	36.00	37.00	0.712	0.619
Final weight (g)	41.00	40.00	42.00	41.00	43.00	0.501	0.386
Cardiac index (%)	0.53	0.46	0.47	0.53	0.57	0.020	0.330
Hepatic index (%)	4.47 ^Bc^	5.25 ^Aa^	4.98 ^ABab^	4.83 ^ABbc^	4.53 ^Bc^	0.072	0.001
Spleen index (%)	0.30 ^b^	0.40 ^a^	0.37 ^ab^	0.38 ^a^	0.41	0.011	0.028
Pulmonary index (%)	0.67	0.79	0.74	0.73	0.72	0.026	0.570
Renal index (%)	1.40	1.65	1.51	1.42	1.45	0.03	0.065

Note: Within each row, values with different superscript letters indicate statistically significant differences. Lowercase letters (a, b, c) denote significant differences at *p* < 0.05; uppercase letters (A, B) denote highly significant differences at *p* < 0.01. Groups sharing at least one identical letter (e.g., both contain “a”, or both contain “B”) are not significantly different from each other; groups sharing no common letter are significantly different at the corresponding threshold.

**Table 3:** The Effects of Different Compound Preparations on the Ileal Morphology of LPS-Challenged Mice.

Items	Groups					SEM	*p*
	C (*n* = 10)	LPS (*n* = 10)	PB (*n* = 10)	AO (*n* = 10)	PB/AO (*n* = 10)		
Villus length/μm	301	173	216	254	270	39	0.205
Crypt depth/μm	141 ^Cc^	265 ^Aa^	151 ^BCc^	190 ^Bb^	167 ^BCbc^	11	<0.001
V/C	2.13 ^Aa^	0.65 ^Bc^	1.43 ^ABb^	1.34 ^ABb^	1.62 ^Aab^	0.24	0.004

Note: Within each row, values with different superscript letters indicate statistically significant differences. Lowercase letters (a, b, c) denote significant differences at *p* < 0.05; uppercase letters (A, B, C) denote highly significant differences at *p* < 0.01. Groups sharing at least one identical letter (e.g., both contain “a”, or both contain “B”) are not significantly different from each other; groups sharing no common letter are significantly different at the corresponding threshold.

**Table 4:** The Effects of Different Compound Preparations on Serum Biochemical Indices of LPS-Challenged Mice.

Items	Groups					SEM	*p*
	C (*n* = 10)	LPS (*n* = 10)	PB (*n* = 10)	AO (*n* = 10)	PB/AO (*n* = 10)		
TP (g/L)	55.67	51.18	52.94	50.25	53.17	0.830	0.294
TC (mmol/L)	1.64	1.43	1.81	2.08	1.76	0.101	0.335
TG (mmol/L)	0.88	1.74	1.92	1.81	2.08	0.153	0.084
BUN (mg/dL)	21.55 ^Bc^	56.86 ^Aa^	43.35 ^Ab^	28.2 ^Bc^	44.53 ^Ab^	2.808	<0.001
GLU (mmol/L)	6.86 ^Aa^	5.24 ^Bb^	5.15 ^Bb^	6.26 ^Aa^	5.01 ^Bb^	0.199	0.001

Note: Within each row, values with different superscript letters indicate statistically significant differences. Lowercase letters (a, b, c) denote significant differences at *p* < 0.05; uppercase letters (A, B) denote highly significant differences at *p* < 0.01. Groups sharing at least one identical letter (e.g., both contain “a”, or both contain “B”) are not significantly different from each other; groups sharing no common letter are significantly different at the corresponding threshold.

**Table 5:** Effects of Different Compound Preparations on Antioxidant and Immune Indexes of LPS-Challenged Mice.

Items	Groups					SEM	*p*
	C (*n* = 10)	LPS (*n* = 10)	PB (*n* = 10)	AO (*n* = 10)	PB/AO (*n* = 10)		
SOD (U/mg)	26.84 ^Aa^	14.59 ^Ce^	18.90 ^Bd^	21.63 ^Bc^	24.72 ^Ab^	0.807	<0.001
GSH-Px (U/mg)	82.40 ^Aa^	54.54 ^Ce^	59.46 ^Cd^	72.37 ^Bc^	77.77 ^ABb^	1.922	<0.001
T-AOC (U/mg)	4.34 ^Aa^	2.87 ^Cc^	3.15 ^BCbc^	3.40 ^Bb^	4.04 ^Aa^	0.111	<0.001
MDA (nmol/mg)	0.78 ^Ee^	2.94 ^Aa^	2.47 ^Bb^	2.11 ^Cc^	1.24 ^Dd^	0.149	<0.001
TNFα (pg/mg)	4.68 ^Ee^	9.45 ^Aa^	8.55 ^ABb^	7.8 ^Bc^	5.83 ^Cd^	0.320	<0.001
IL-1β (pg/mg)	1.57 ^Cc^	2.61 ^Aa^	2.54 ^Aa^	2.23 ^ABb^	2.02 ^Bb^	0.082	<0.001
IL-6 (pg/mg)	7.71 ^Ed^	15.11 ^Aa^	14.31 ^ABa^	13.25 ^Bb^	9.90 ^Cc^	0.505	<0.001
DAO (U/mg)	1.03 ^Cd^	2.10 ^Aa^	1.85 ^AaBb^	1.64 ^ABbc^	1.40 ^BCc^	0.084	<0.001

Note: Within each row, values with different superscript letters indicate statistically significant differences. Lowercase letters (a, b, c, d, e) denote significant differences at *p* < 0.05; uppercase letters (A, B, C, D, E) denote highly significant differences at *p* < 0.01. Groups sharing at least one identical letter (e.g., both contain “a”, or both contain “B”) are not significantly different from each other; groups sharing no common letter are significantly different at the corresponding threshold.

**Table 6:** The Effects of Different Compound Preparations on Ileal Microbiota α-Diversity of LPS-Challenged Mice.

Items	Groups					SEM	*p*
	C	LPS	PB	AO	PB/AO		
Chao1	140.68 ^a^	95.43 ^ab^	38.93 ^b^	62.54 ^b^	104.80 ^ab^	12.048	0.061
Observed_features	138.00 ^a^	89.67 ^ab^	38.67 ^b^	60.17 ^b^	102.50 ^ab^	11.500	0.049
Pielou_e	0.37	0.37	0.44	0.33	0.45	0.021	0.189
Dominance	0.37	0.41	0.34	0.45	0.26	0.030	0.239
Simpson	0.64	0.59	0.66	0.55	0.74	0.032	0.239
Shannon	2.63	2.26	2.30	1.86	2.92	0.157	0.196

Note: Within each row, values with different superscript letters indicate statistically significant differences. Lowercase letters (a, b) denote significant differences at *p* < 0.05. Groups sharing at least one identical letter (e.g., both contain “a”) are not significantly different from each other; groups sharing no common letter are significantly different at the corresponding threshold.

**Table 7:** The Effects of Different Compound Preparations on Metabolites in Mouse Ileal Chyme.

Items	Groups					SEM	*p*
	C	LPS	PB	AO	PB/AO		
Acetate (μm/g)	14.48	11.09	3.23	4.57	5.86	1.880	0.236
Propionate (μm/g)	0.84	2.00	0.18	0.52	0.19	0.322	0.368
Butyrate (μm/g)	1.54	0.15	0.07	0.07	0.08	0.276	0.326

Note: Data are presented as mean ± SEM (n = 10 per group).

## Data Availability

All of the data is contained within the article.
